# Systemic skewing of peripheral blood leukocyte composition in neurofibromatosis type 1

**DOI:** 10.3389/fimmu.2026.1849927

**Published:** 2026-06-30

**Authors:** Yoshimasa Nobeyama, Satoko Imai, Minako Ogawa-Tominaga, Akihiko Asahina

**Affiliations:** Department of Dermatology, The Jikei University School of Medicine, Tokyo, Japan

**Keywords:** basophil, eosinophil, lymphocyte, monocyte, neutrophil, NF1, type 2 immunity

## Abstract

**Background:**

The present study aimed to characterize neurofibromatosis type 1 (NF1)-associated alterations in peripheral white blood cell differentials, focusing on overall patterns and sex-related differences.

**Methods:**

We analyzed 225 Japanese patients with NF1 (78 males, 147 females) and 87 controls without NF1 (50 males, 37 females). Within each sex, NF1 and control groups were age-matched. Z-scores were calculated to normalize for sex-specific variation.

**Results:**

In patients with NF1, Z-scores for the percentages of neutrophils, monocytes, and basophils were increased, whereas those for lymphocytes and eosinophils were decreased. These trends were consistent across both sexes, indicating robust disease-associated alterations. Additional sex-related differences were observed: males showed greater neutrophil increases and more pronounced lymphocyte reductions, whereas females exhibited greater monocyte increases and more marked eosinophil reductions.

**Conclusion:**

NF1 is associated with systemic skewing of peripheral immune cell composition, characterized by expansion of the non–type 2/innate component, relative uncoupling of the type 2–associated component, and reduction of the lymphoid component. These findings provide a conceptual basis for understanding NF1 pathobiology, disease heterogeneity, and sex-related differences, including tumor-associated immune environments.

## Introduction

Neurofibromatosis type 1 (NF1) is an autosomal dominant disorder caused by germline mutations in the NF1 gene. Its gene product, neurofibromin, is best known as a negative regulator of rat sarcoma virus (RAS) signaling, but also modulates additional signaling pathways, thereby contributing to diverse cellular processes. Clinically, NF1 is characterized by a broad spectrum of manifestations affecting multiple organ systems, including cutaneous, neural, and skeletal features (1).

Accumulating evidence indicates that inflammatory processes are involved in the pathogenesis of these manifestations. Neurofibroma development is associated with a tumor microenvironment enriched in mast cells and macrophages under conditions of *NF1* haploinsufficiency (2, 3). In addition, inflammation-associated gene signatures induced by nerve injury have been identified in NF1 model systems, suggesting that inflammatory responses contribute to tumor growth (4). Beyond tumor biology, neurofibromin deficiency has been linked to abnormal early inflammatory responses, such as excessive macrophage accumulation during bone repair, leading to impaired callus formation and non-union (5). Collectively, these findings support the concept that NF1 is associated with dysregulated inflammatory processes across multiple tissues.

In general, peripheral leukocyte differentials provide an accessible and clinically translatable readout of systemic immune status and inflammatory balance. Consequently, they have been utilized as biomarkers across a diverse range of inflammatory and neoplastic disorders. Given that NF1 pathogenesis is potentially associated with immune processes, peripheral leukocyte differentials may offer valuable clues to understanding disease development mechanisms and estimating the risks of specific symptoms.

Numerous studies have suggested sex dimorphism in both innate and adaptive immune responses within the general population (6). Sex chromosome genes and sex hormones are well-known to regulate these differential immune responses, consequently influencing disease phenotypes beyond inflammatory disorders (7, 8). In that context, the impact of sex-specific immune differences in NF1 warrants closer investigation.

While genotype–phenotype correlations have been reported in specific NF1 cohorts (9–11), sex-related differences in clinical manifestations have become more widely recognized. Although the prevalence of optic pathway glioma does not differ between males and females, the risk of visual impairment is approximately threefold higher in female patients (12). Male patients exhibit a higher risk of spatial cognitive impairment and learning disabilities, whereas female patients experience a greater psychological burden related to disfigurement (13–15). However, it remains unclear whether such sex-related differences extend to fundamental biological parameters, including immune cell composition.

Emerging evidence indicates that NF1 is associated with alterations in peripheral blood cell counts. Our previous study suggested both shared and sex-specific patterns, including increased neutrophil, monocyte, and basophil counts in males and decreased lymphocyte counts in both sexes (16). Although sex differences remain a subject for further investigation, a direct comparison of raw values is inappropriate due to inherent physiological dimorphism. Consequently, it remains unclear whether these observations reflect NF1-specific sex differences or systemic alterations independent of sex.

In addition to analyzing the raw data, we addressed this issue by converting the hematological data into Z-scores to quantify deviations from sex-specific reference values. This approach enables standardized assessment of NF1-associated alterations by accounting for physiological differences between sexes and allows comparison of deviation magnitude across groups. Using this framework, the present study aimed to characterize NF1-associated alterations in peripheral differential white blood cell (WBC) counts and to determine whether these alterations exhibit sex-related differences.

## Materials and methods

### Patients

The ethics committee of The Jikei University School of Medicine, Tokyo, Japan, approved the study protocol, and informed consent was obtained in the form of opt-out for all participants.

Patients were enrolled based on the following inclusion criteria: i) referral to The Jikei University School of Medicine between January 2018 to February 2025 for the resection of neurofibromas (for patients with NF1), or between January 2017 and December 2018 in control reference population for the resection of benign cutaneous tumors including lipoma, atheroma, or melanocytic nevus without clinical features suggestive of NF1 (for the control reference population); and the implementation of routine preoperative blood testing for all eligible individuals; ii) for patients with NF1, fulfillment of the revised diagnostic criteria (17); iii) for both the control and NF1 groups, availability of blood test results from preoperative evaluations performed within 6 weeks prior to surgery; iv) for both the control and patient groups, absence of acute or chronic systemic illnesses, including hematological, inflammatory, autoimmune, and malignant diseases, at the time of evaluation; and v) for both the control and patient groups, absence of prior exposure to medications known to potentially have a profound impact on hematological findings, including molecular targeted agents, immune checkpoint inhibitors, cytotoxic chemotherapeutic agents, immunosuppressants, and glucocorticoids.

Age matching between NF1 and control groups was performed within each sex using 10-year age strata, allowing a maximum deviation of ±5% in proportional distribution across strata. Consequently, we retrospectively analyzed 225 Japanese patients with NF1 (78 males, mean age 50.3 ± 14.4 years; 147 females, mean age 48.4 ± 12.7 years) and 87 Japanese controls without NF1 (50 males, mean age 51.4 ± 13.5 years; 37 females, mean age 47.9 ± 13.7 years). The control group included patients with lipoma (n=45; 28 males, 17 females), atheroma (n=23; 15 males, 8 females), and melanocytic nevus (n=19; 7 males, 12 females) (**Supplementary Table 1**).

The characteristics of WBC counts and differentials for the control reference and NF1 patient groups are presented in **Supplementary Tables 2**, **3**, respectively. Clinical characteristics, raw values for total WBC counts and differential percentages, and their corresponding Z-scores for each patient with NF1, as well as mean values and standard deviations for each parameter derived from the control reference population, are provided in **Supplementary Table 4**.

### Examination

Symptom severity was graded according to the DNB severity classification system. Each D-class was defined as follows: D1, pigmented macules and a few neurofibromas; D2, pigmented macules and many neurofibromas; D3, numerous neurofibromas (>1000 in number, >1 cm in size); D4, severe plexiform neurofibromas or a malignant peripheral nerve sheath tumor. Each N-class was defined as follows: N0, no neurological symptoms; N1, neurological symptoms (e.g., paralysis or pain) and/or abnormal neurological findings; N2, severe or progressive neurological symptoms. N-class was determined based on the following neurological findings; medical history taken from the patients/parents and neurological objective findings including body length, body weight, head measurement, developmental index, mental status, motor function and balance, sensory examination, newborn and infant reflexes, muscle stretch reflexes, and evaluation of the cranial nerves. Each B-class was defined as follows: B0, no bone lesions; B1, a mild or moderate bone lesion (deformity in the spine or extremities that does not require treatment); B2, a severe bone lesion (dystrophic type or spine deformity that requires surgery (e.g., scoliosis or kyphosis), severe bone deformity in the extremities (e.g., pseudarthrosis and fracture), or a defect of the skull or facial bone.

Based on the D-class criteria, patients were categorized as follows: D1 (0 males, 7 females), D2 (28 males, 44 females), D3 (30 males, 46 females), and D4 (20 males, 50 females). Similarly, under the N-class criteria, patients were distributed into N0 (41 males, 65 females), N1 (36 males, 75 females), and N2 (1 male, 7 females). For the B-class classification, 50 males and 101 females were classified as B0, 19 males and 29 females as B1, and 9 males and 17 females as B2. No significant sex differences were observed in the distribution of D-, N-, and B-classes (chi-squared test; data not shown).

Biochemical blood analysis was performed with an automatic analyzer LABOSPECT 008 α (Hitachi High-Tech, Tokyo, Japan). Blood samples for both the control and patient groups were collected during the daytime (between 9:00 AM and 4:00 PM) at the outpatient clinic within 6 weeks prior to surgery for the purpose of a preoperative check. Because these were elective surgeries, the preoperative evaluations were conducted during periods free of acute illness.

### Calculation and statistical analysis

Z-scores were calculated by subtracting the control mean from each individual NF1 value and dividing the result by the control standard deviation. To evaluate differences in quantitative values, the Mann-Whitney U test was employed for sex-related comparisons, while the Kruskal-Wallis test was used to assess differences across DNB classifications. The degree of Z-score deviation from zero was determined using the one-sample Wilcoxon signed-rank test. To evaluate sex differences in the distribution of D-, N-, and B-classes, a chi-squared test was performed. The above statistical analysis was performed using SPSS version 22 (IBM Corp., Armonk, NY). The Benjamini-Hochberg procedure was applied to control the false discovery rate for all analyses using R (version 4.6.0; R Foundation for Statistical Computing, Vienna, Austria). A *P*-value < 0.05 was considered statistically significant.

## Results

To ensure that our control cohort is appropriate as a reference standard, sex-related differences were examined. The analysis revealed findings highly consistent with previous physiological reports (**Supplementary Table 5**): WBC counts and most leukocyte subsets exhibited minor or negligible differences between sexes (18), with the notable exception that monocyte levels were naturally higher in males (19, 20). These findings confirm that our control data accurately reflect expected physiological baselines rather than capturing any cohort-specific artifacts, validating its reliability as a reference.

In the raw data analysis, significant differences were detected in five out of six parameters for the pooled data, three for males, and five for females (**Supplementary Table 6**). However, a simple pooled analysis carries a substantial risk of statistical bias and can obscure true sex-specific trends in the combined data, because the results are heavily weighted toward the group with the larger sample size (78 males vs. 147 females in the patient group; 50 males vs. 37 females in the control group). Furthermore, such sample size discrepancies confound direct comparisons of statistical power between sexes. To overcome these limitations and detect NF1-specific leukocytic characteristics on an equal scale across males, females, and the combined cohort, we employed a Z-score-based analysis to eliminate biases arising from both physiological sex dimorphism and sample size disparities.

**Figure 1:**
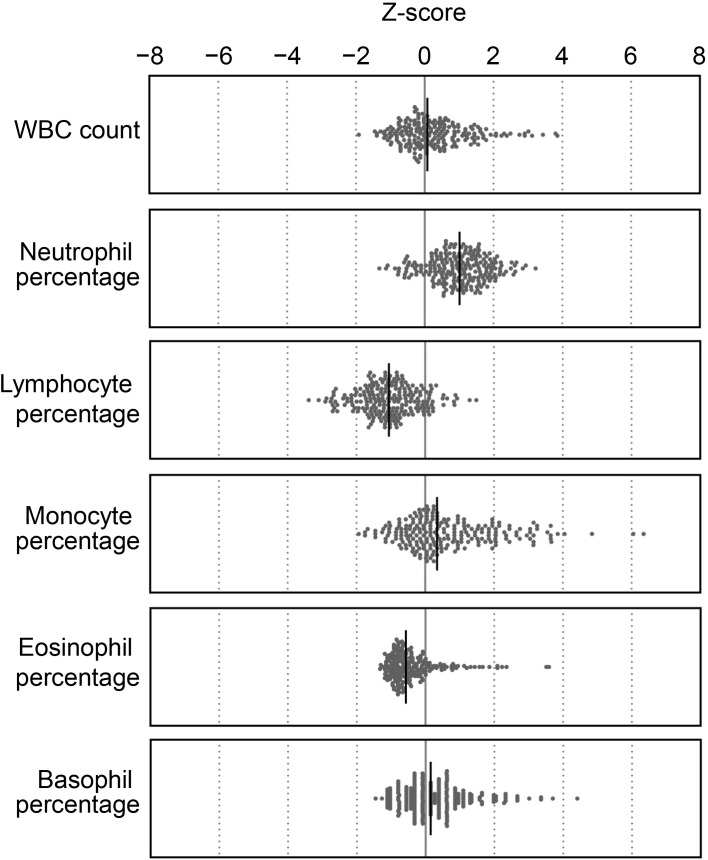
Distribution of Z-scores in overall cohort. The vertical and horizontal axes represent each parameter and the Z-score, respectively. In the beeswarm plots, dots and vertical line represent individual values and median values, respectively.

In the overall cohort, one-sample Wilcoxon signed-rank tests demonstrated that Z-scores for total WBC counts and the percentages of neutrophils, monocytes, and basophils were significantly above zero (all *P* < 0.05), indicating a systemic upward shift in these parameters (**Table 1**; **Figure 1**). Conversely, Z-scores for lymphocyte and eosinophil percentages were significantly below zero (all *P* < 0.05), reflecting a downward shift. In the Z-scores for WBC counts and differentials, no significant differences were observed across DNB-classifications (**Supplementary Table 7**).

**Table 1:** Z-score analysis of leukocyte differentials relative to zero baseline.

WBC count/ leukocyte differential	Median value	*P*-value
WBC count	0.079	0.004
Neutrophil percentage	1.009	<0.001
Lymphocyte percentage	−1.057	<0.001
Monocyte percentage	0.345	<0.001
Eosinophil percentage	−0.571	<0.001
Basophil percentage	0.147	0.001

WBC, white blood cell.

**Figure 2:**
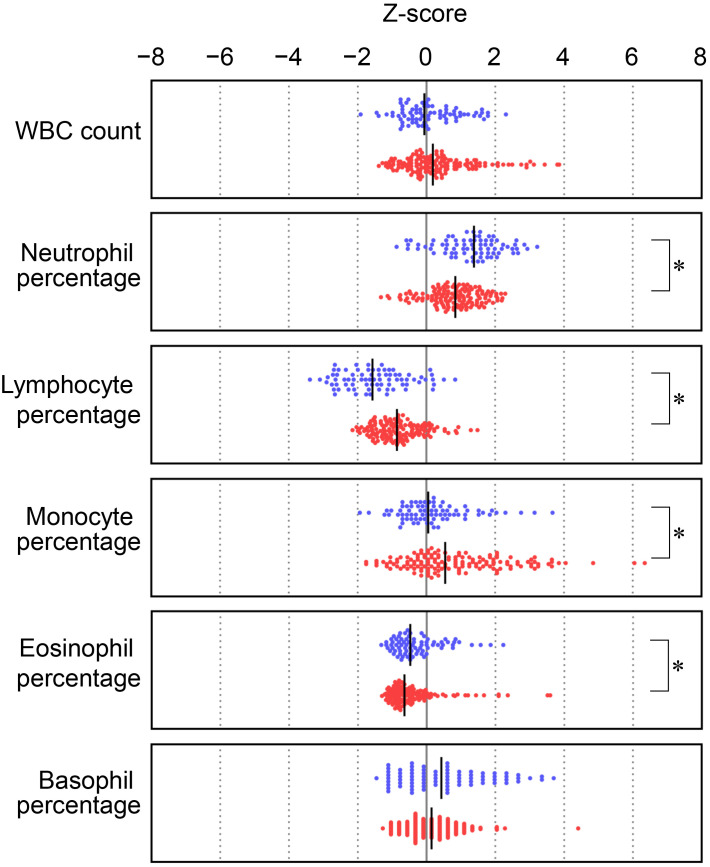
Distribution of Z-scores by sex. The vertical and horizontal axes represent each parameter and the Z-score, respectively. In the beeswarm plots, dots and vertical line represent individual values and median values, respectively. Blue and red dots indicate male and female data, respectively. Asterisks denote significant differences between male and female data.

In sex-stratified analyses, Z-score based analyses showed that both male and female patients exhibited systemic shifts in leukocyte parameters relative to reference values. In both sexes, Z-scores for the percentages of neutrophils and basophils were significantly above zero (one-sample Wilcoxon signed-rank tests, all *P* < 0.05) (**Table 2**; **Figure 2**). Conversely, lymphocyte and eosinophil percentages showed significant downward shifts, with Z-scores significantly below zero (one-sample Wilcoxon signed-rank tests, all *P* < 0.05). In addition, Z-scores for total WBC counts and monocyte percentages were significantly above zero in females (one-sample Wilcoxon signed-rank test, P < 0.05), whereas no significant differences were observed in males. Notably, the two sexes exhibited a similar trend in monocyte percentages, whereas their trends for total WBC counts were divergent. While the direct analysis of raw data largely supported the overall trends (**Supplementary Table 6**), the Z-score-based analysis exhibited enhanced sensitivity. Through this approach, subtle deviations in male eosinophil and female basophil percentages, which were obscured in the raw data, were captured as statistically significant.

**Table 2:** Z-score analysis of leukocyte differentials by sex relative to zero baseline.

WBC count/ leukocyte differential	Male (n = 78)		Female (n = 147)	
	Median value	*P*-value	Median value	*P*-value
WBC count	−0.064	0.744	0.184	0.001
Neutrophil percentage	1.384	<0.001	0.841	<0.001
Lymphocyte percentage	−1.569	<0.001	−0.855	<0.001
Monocyte percentage	0.052	0.348	0.549	<0.001
Eosinophil percentage	−0.481	<0.001	−0.648	<0.001
Basophil percentage	0.433	0.004	0.147	0.030

WBC, white blood cell.

Regarding inter-sex comparisons, analysis of raw data initially showed significant differences in monocyte and eosinophil percentages between male and female NF1 patients (Mann-Whitney U test; **Supplementary Table 8**). However, raw data comparisons are inherently confounded by baseline physiological differences between sexes, which can mask NF1-specific characteristics. To overcome this limitation, we utilized a Z-score-based analysis for a combined evaluation. Consequently, Mann-Whitney U tests on the Z-scores revealed significant differences in neutrophil, lymphocyte, monocyte, and eosinophil percentages between the sexes (**Table 3**, **Figure 2**). Specifically, male patients exhibited significantly greater positive deviations in neutrophil percentages (*P* < 0.001) and more severe negative deviations in lymphocyte percentages (*P* < 0.001) compared to females. In contrast, female patients demonstrated more pronounced positive deviations in monocyte percentages (*P* = 0.001) and more marked negative deviations in eosinophil percentages (*P* = 0.046) than their male counterparts.

**Table 3:** Sex differences in Z-scores for leukocyte differentials.

WBC count/ leukocyte differential	Median value		
	Male (n = 78)	Female (n = 147)	*P*-value
WBC count	−0.064	0.184	0.101
Neutrophil percentage	1.384	0.841	<0.001
Lymphocyte percentage	−1.569	−0.855	<0.001
Monocyte percentage	0.052	0.549	0.001
Eosinophil percentage	−0.481	−0.648	0.046
Basophil percentage	0.433	0.147	0.101

WBC, white blood cell.

## Discussion

The present study demonstrates that i) differential WBC counts are systemically skewed in patients with NF1, and ii) the magnitude and pattern of this skewing differ between males and females, collectively indicating systemic immune and inflammatory dysregulation in NF1.

### Total WBC counts in NF1

In the overall NF1 cohort, Z-scores for total WBC counts were modestly but significantly elevated, primarily driven by increases in females. However, no significant sex difference was observed, suggesting a small effect size and limited sex specificity. Furthermore, no significant differences were observed across other DNB classifications. These findings suggest that total WBC counts alone do not adequately capture NF1-associated alterations and underscore the importance of differential analysis as a more sensitive indicator of systemic immune and inflammatory status.

### Expansion of the non–type 2/innate component in NF1

A previous study has suggested increased neutrophil and monocyte counts in male patients with NF1 (16). In the present study, Z-scores for neutrophil percentages were significantly elevated in both sexes as well as in the overall cohort. However, Z-scores of monocyte percentages showed a significant increase in the overall cohort and in females, but not in males. Based on these experimental insights, the lack of statistical significance in monocyte percentages among male patients may be attributed to baseline physiological differences. Historically, males exhibit higher physiological monocyte levels than females; consequently, the inherently elevated baseline in the male control group may have masked the subtle statistical impact of *NF1* haploinsufficiency in this sex. Furthermore, because monocyte counts are susceptible to age-related physiological fluctuations, raw data comparisons can be confounded by differences in age distribution. To address this, the present study aligned the age distributions between the NF1 and control groups within a ±5% deviation across 10-year strata and evaluated the data using Z-scores to control for age-associated confounding effects. Therefore, our findings likely provide a more accurate and robust reflection of the biological status, minimizing potential age-related bias. Nevertheless, a clear upward trend in monocyte percentages was still observable in males with NF1, suggesting that the underlying biological mechanism remains active across both sexes despite the lack of statistical significance.

Supporting this hypothesis, a plausible explanation for the overall increase in neutrophil and monocyte percentages is an intrinsic effect of *NF1* haploinsufficiency on hematopoietic stem and progenitor cells. Neurofibromin is best known as a negative regulator of RAS signaling; however, it also modulates additional signaling pathways, and its deficiency results in dysregulated intracellular signaling, including constitutive activation of RAS-dependent pathways. Experimental studies have shown that *NF1* loss enhances progenitor sensitivity to cytokines such as granulocyte–macrophage colony-stimulating factor, promoting expansion of granulocyte–macrophage progenitors and resulting in myeloproliferative features (21).

Importantly, neurofibromas contain abundant macrophages derived from circulating monocytes (22, 23). These findings support the concept that NF1 is associated with a systemic shift toward innate and inflammatory activation, linking peripheral blood findings with tissue-level inflammatory processes.

### Uncoupling of the type 2–associated component in NF1

In contrast to the expansion of the non–type 2/innate component, the type 2–associated component exhibited a relative uncoupling, characterized by opposing changes in eosinophil and basophil proportions in the peripheral blood.

Z-scores for eosinophil percentages were significantly decreased in both sexes, with a more pronounced reduction in females. The finding suggests attenuation of type 2–associated effector responses, particularly in female patients. This observation is consistent with clinical data indicating a higher risk of visual impairment in female patients with NF1-associated optic pathway glioma (12). Emerging evidence indicates that type 2 immune responses can exert glioma-suppressive effects, including through modulation of microglial activity and chemokine signaling (24). Reduced type 2 effector activity, as reflected by eosinophil depletion, may therefore contribute to sex differences in glioma progression in NF1.

Notably, eosinophil reduction occurred in a pattern distinct from the expansion of other myeloid-lineage cells, suggesting differential regulation within the myeloid compartment. Eosinophil differentiation is closely associated with type 2 cytokines such as interleukin-5 (25) and may be regulated independently of granulocyte–macrophage lineage expansion. Thus, the decrease in eosinophils may reflect a distinct regulatory process, such as attenuation of type 2–associated immunity or altered trafficking to peripheral tissues.

In contrast, a previous study has suggested increased basophil counts in male patients with NF1 (16). In the present study, by virtue of our well-balanced age matching and z-score transformation which enhanced statistical accuracy, Z-scores for basophil percentages were significantly elevated in both sexes as well as in the overall cohort, indicating a consistent increase independent of sex. Although basophils and mast cells differ in localization and developmental origin, they share key functional properties (26). Given the well-recognized positive correlation between eosinophil and basophil counts in peripheral blood, the increase in basophils, in contrast to eosinophil reduction, suggests uncoupled regulation within the type 2–associated compartment (27). This pattern may reflect dysregulation of the basophil–mast cell lineage in NF1, potentially driven by altered intracellular signaling, including enhanced RAS-dependent pathways, as well as changes in cytokine responsiveness (28).

Collectively, these findings indicate relative uncoupling within the type 2–associated component, characterized by reduced eosinophil-mediated effector responses and increased basophil-associated elements, further highlighting an imbalance in the systemic inflammatory milieu.

### Reduction of the lymphoid component in NF1

Consistent with a previous report (16), Z-scores for lymphocyte percentage were significantly decreased in both sexes as well as in the overall cohort. One potential explanation is altered immune cell trafficking, resulting in the redistribution of lymphocytes from the circulation to peripheral tissues (29). However, given the lack of strong associations with clinical phenotypes, this mechanism may be less likely. Alternatively, dysregulated intracellular signaling, including aberrant RAS-dependent pathways, may impair lymphocyte development and disrupt lineage balance within the hematopoietic system (30).

### Integrated immune–inflammatory features in NF1

**Figure 3:**
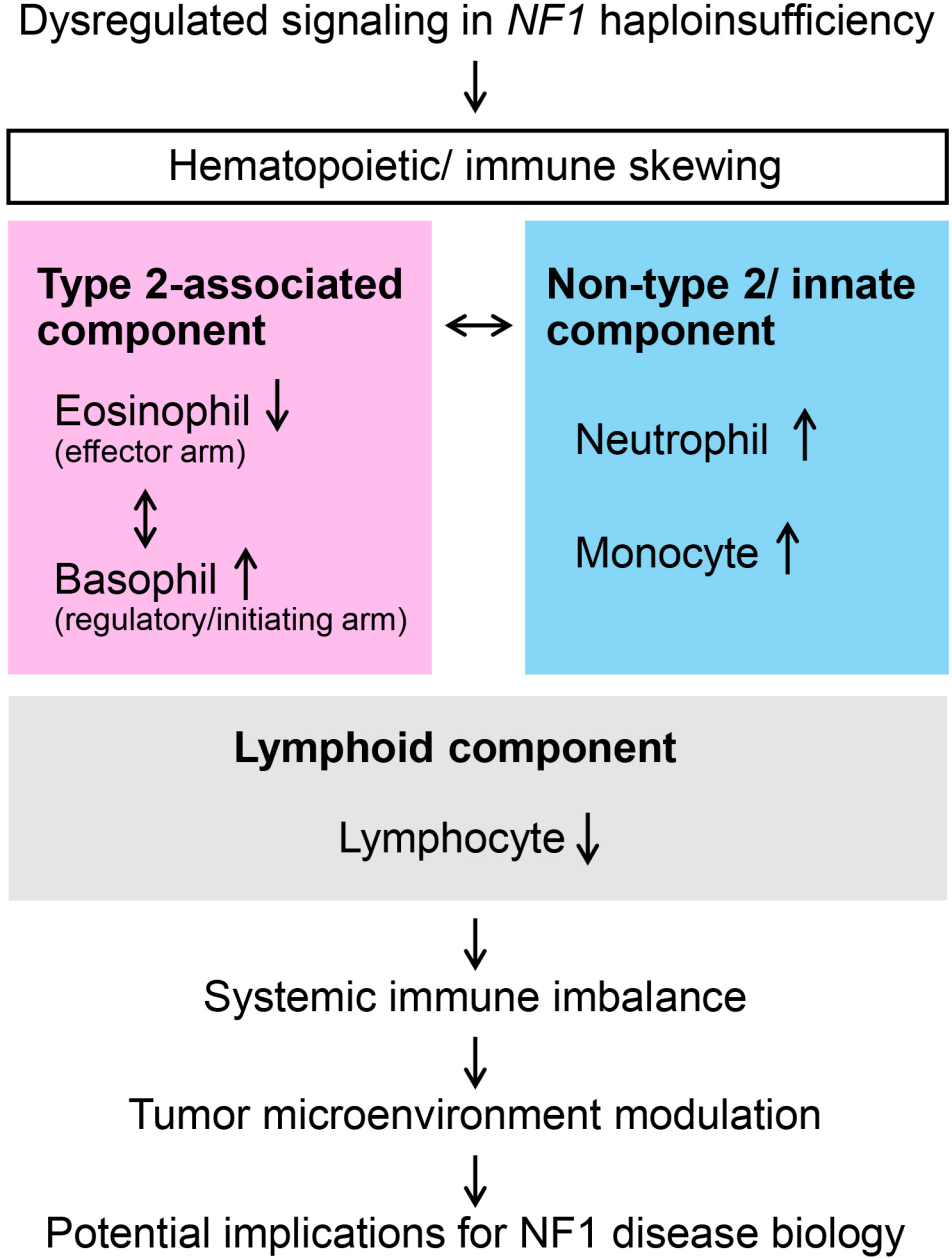
Schematic representation of systemic immune component skewing in NF1. *NF1* haploinsufficiency leads to dysregulated intracellular signaling, including enhanced RAS-dependent pathways, resulting in systemic immune component skewing. The type 2–associated component shows relative uncoupling with attenuated effector activity, characterized by decreased eosinophils and increased basophils. The non–type 2/innate component is expanded, as reflected by increased neutrophils and monocytes, and exhibits a reciprocal relationship with the type 2–associated component. The relationship with the lymphoid compartment remains to be determined, suggesting a potential axis of immune imbalance in NF1.

Collectively, these results indicate that NF1 is characterized by systemic skewing of immune cell composition, specifically: i) expansion of the non–type 2/innate component, ii) relative uncoupling of the type 2–associated component, and iii) reduction of the lymphoid component (**Figure 3**). The reciprocal relationship between the type 2–associated and non–type 2/innate components supports coordinated skewing of inflammatory responses within the immune system. In this context, the interplay between these component-specific alterations and the shift in the lymphoid compartment warrants further investigation. Overall, these findings support the concept that peripheral blood leukocyte composition reflects systemic inflammatory processes in NF1.

Notably, the observed skewing of peripheral leukocyte composition did not show clear concordance with clinical phenotypic classifications, such as the DNB categories. If these alterations were merely secondary to established disease manifestations, a closer association with clinical phenotypes would be expected. The lack of such correspondence suggests that immune component skewing may represent a more fundamental, system-level feature of NF1 biology rather than a downstream consequence of specific clinical manifestations. This raises the possibility that immune dysregulation contributes to shaping disease heterogeneity rather than simply reflecting it. These observations also suggest that peripheral leukocyte profiles may serve as accessible biomarkers for systemic immune dysregulation and disease stratification in NF1.

### Sex dimorphism of peripheral leukocyte composition in NF1

Generally, males and females exhibit distinct disease susceptibilities and immune response magnitudes, a phenomenon known as sexual dimorphism in immunity (6, 31). Nevertheless, in the healthy general population, total WBC counts and most leukocyte subsets exhibit minor or negligible sex-related differences (18), with the notable exception that monocytes tend to be higher in males (19, 20). The control reference group in our study aligned with these established trends, validating that our overall control cohort lacked major physiological bias and served as an appropriate baseline.

Crucially, however, even these subtle baseline variations, combined with profound functional sex dimorphism, can distort statistical analyses when patient and control cohorts suffer from severe sample size imbalances. Under such conditions, a simple pooled analysis of raw data risks introducing statistical artifacts and masking true sex-specific trends. By utilizing a Z-score-based analysis to overcome these limitations and standardize the datasets, the present study successfully revealed distinct, sex-specific leukocytic deviations in NF1 patients. These findings suggest that peripheral blood immunocompetent cells in NF1 patients may be more susceptible to mechanisms linked to sex hormones and X-linked immune regulation than those in healthy individuals.

Regarding functional subpopulations, eosinophil depletion in females, rather than in males, appeared to drive abnormalities within the type 2-associated component. Within the non-type 2/innate component, these aberrations were underlain by an elevated neutrophil percentage in males and an increased monocyte percentage in females. Furthermore, lymphoid component abnormalities were more pronounced in males. Collectively, these findings suggest that sex dimorphism in non-type 2/innate immunity, coupled with NF1-biased neutrophilic inflammation, is more prominent in male patients than in female patients, compatible with trends in other conditions including autoinflammatory disorders (32). Crucially, the mutually complementary changes, increased neutrophil percentages in NF1 males and increased monocyte percentages in NF1 females, may collectively skew the non-type 2/innate component. This compensatory shift could explain the lack of sex differences in most NF1 phenotypes, with the notable exception of eosinophil depletion in females, which aligns with the higher tendency of optic pathway glioma occurrence. Further studies are warranted to clarify how these sex-specific leukocyte deviations in NF1 patients translate into actual functional and clinical phenotypes.

### Limitations

The present study has several limitations. First, owing to the retrospective nature of the study, information regarding some minor medications that might subtly influence WBC counts and leukocyte differentials could be incomplete, preventing complete control over all pharmacological variables. Second, the sample size was not determined by formal power calculation, and limited statistical power for certain analyses cannot be excluded. Third, the modest sample size and sex imbalance may introduce bias related to physiological differences. Fourth, D-classification was not consistently assessed using whole-body magnetic resonance imaging, which may have led to underestimation in some patients.

### Conclusion

The present study demonstrates systemic skewing of peripheral leukocyte composition in NF1. The changes reflect coordinated skewing of inflammatory responses within the immune system and may represent a fundamental feature of NF1 biology. This framework provides a conceptual basis for understanding NF1 pathobiology, disease heterogeneity, and sex-related differences, including tumor-associated immune environments.

## Data Availability

The data analyzed in this study is subject to the following licenses/restrictions: The datasets generated and analyzed during the current study are available from the corresponding author upon reasonable request. Individual-level data for the control reference population are not publicly available as they are part of a separate article currently under peer review. To avoid duplication of published material, the present study presents only aggregated data, including sample sizes, means, standard deviations, and mean age stratified by sex. Access to the full individual-level dataset may be granted upon reasonable request once the related article has been published. Requests to access these datasets should be directed to Yoshimasa Nobeyama, nobederm@jikei.ac.jp.

## Supplementary material

Supplementary Figure 1 Distribution of raw data in the control population and patients with NF1. The vertical and horizontal axes represent each parameter and the raw data value, respectively. In the beeswarm plots, dots and vertical line represent individual values and median values, respectively. Gray and black dots indicate control and NF1 patient data, respectively. Asterisks denote significant differences between male and female data. **(A)** sex-combined, **(B)** male, and **(C)** female data.
