# Supplementary figures and images for: Systemic skewing of peripheral blood leukocyte composition in neurofibromatosis type 1
Source: Front Immunol. 2026 Jun 30;17:1849927. doi: 10.3389/fimmu.2026.1849927 (PMC13364682; doi:10.3389/fimmu.2026.1849927)

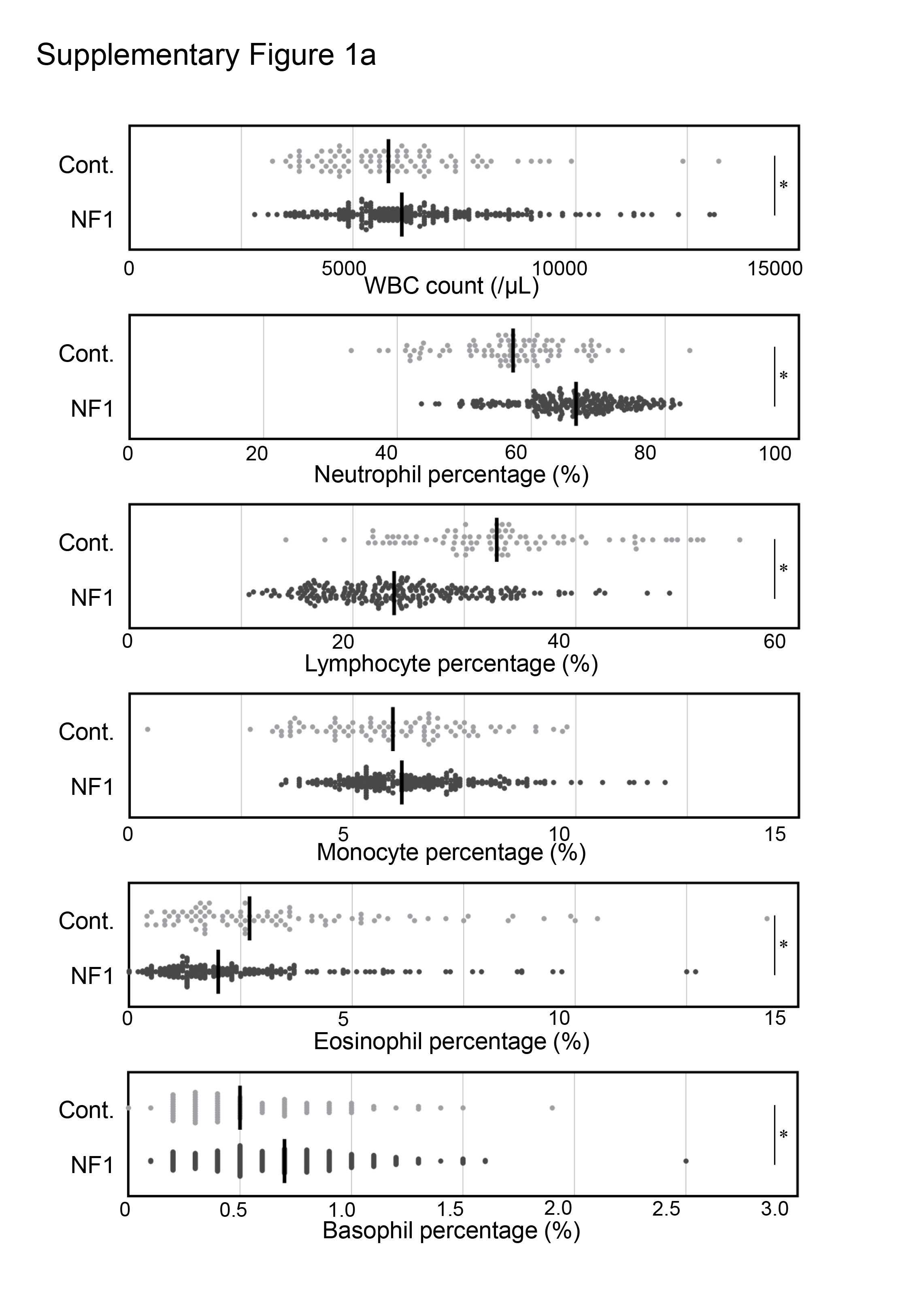

Supplement: Supplementary Figure 1 — Distribution of raw data in the control population and patients with NF1. The vertical and horizontal axes represent each parameter and the raw data value, respectively. In the beeswarm plots, dots and vertical line represent individual values and median values, respectively. Gray and black dots indicate control and NF1 patient data, respectively. Asterisks denote significant differences between male and female data. (A) sex-combined, (B) male, and (C) female data. [file Image1.jpeg]

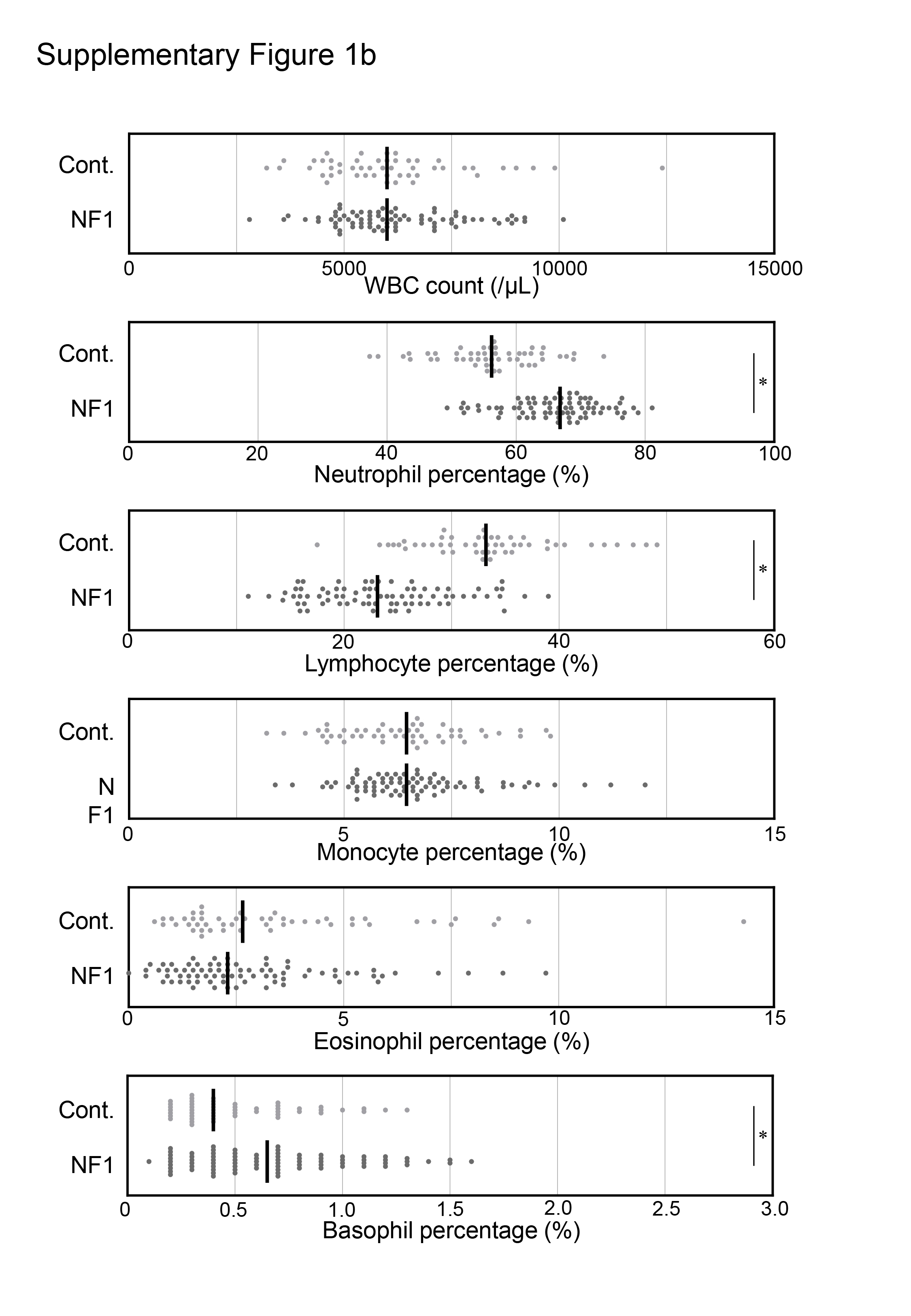

Supplement: Supplementary file 2 [file Image2.jpeg]

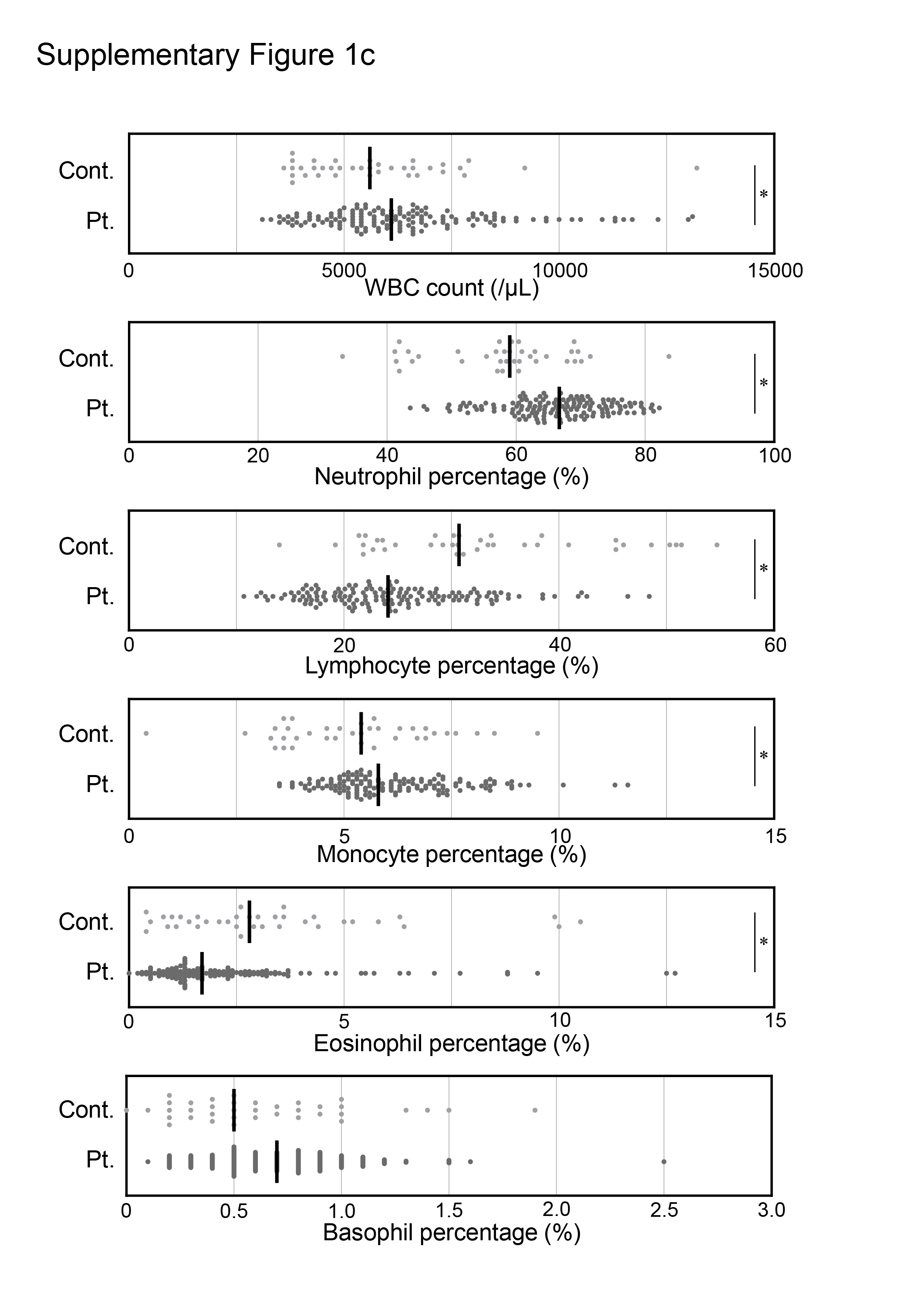

Supplement: Supplementary file 3 [file Image3.jpeg]
