# Supplementary material for: Systemic skewing of peripheral blood leukocyte composition in neurofibromatosis type 1
Source: Front Immunol. 2026 Jun 30;17:1849927. doi: 10.3389/fimmu.2026.1849927 (PMC13364682; doi:10.3389/fimmu.2026.1849927)
Supplement: Supplementary file 5 [file Table2.docx]

**Supplementary Table 2a. Characteristics of leukocyte differentials in the total control group**

Mean value SD Minimum value Maximum value

WBC count (/µL) 5956 1806 3200 13200

Neutrophil percentage (%) 56.75 9.106 33.1 83.7

Lymphocyte percentage (%) 33.25 8.364 14.0 54.7

Monocyte percentage (%) 5.894 1.753 0.4 9.8

Eosinophil percentage (%) 3.516 2.664 0.4 14.3

Basophil percentage (%) 0.572 0.355 0.0 1.9

WBC, white blood cell; SD, standard deviation.

**Supplementary Table 2b. Characteristics of leukocyte differentials in control male subjects**

Mean value SD Minimum value Maximum value

WBC count (/µL) 6110 1730 3200 12400

Neutrophil percentage (%) 56.00 7.783 37.3 73.6

Lymphocyte percentage (%) 33.46 6.603 17.5 49.1

Monocyte percentage (%) 6.370 1.536 3.2 9.8

Eosinophil percentage (%) 3.616 2.735 0.6 14.3

Basophil percentage (%) 0.524 0.291 0.2 1.3

WBC, white blood cell; SD, standard deviation.

**Supplementary Table 2c. Characteristics of leukocyte differentials in control female subjects**

Mean value SD Minimum value Maximum value

WBC count (/µL) 5749 1908 3600 13200

Neutrophil percentage (%) 57.72 10.67 33.1 83.7

Lymphocyte percentage (%) 32.97 10.37 14.0 54.7

Monocyte percentage (%) 5.251 1.842 0.4 9.5

Eosinophil percentage (%) 3.381 2.596 0.4 10.5

Basophil percentage (%) 0.638 0.423 0.0 1.9

WBC, white blood cell; SD, standard deviation.
