# Supplementary material for: Systemic skewing of peripheral blood leukocyte composition in neurofibromatosis type 1
Source: Front Immunol. 2026 Jun 30;17:1849927. doi: 10.3389/fimmu.2026.1849927 (PMC13364682; doi:10.3389/fimmu.2026.1849927)
Supplement: Supplementary file 6 [file Table3.docx]

**Table 3a. Characteristics of leukocyte differentials in the total NF1 group**

Mean value SD Minimum value Maximum value

WBC count (/µL) 6834 1855 2800 13100

Neutrophil percentage (%) 66.20 7.730 43.6 82.2

Lymphocyte percentage (%) 24.24 6.925 10.7 48.4

Monocyte percentage (%) 6.350 1.550 3.4 12.0

Eosinophil percentage (%) 2.507 2.055 0.0 12.7

Basophil percentage (%) 0.696 0.353 0.1 2.5

WBC, white blood cell; SD, standard deviation.

**Table 3b. Characteristics of leukocyte differentials in the male NF1 group**

Mean value SD Minimum value Maximum value

WBC count (/µL) 6259 1476 2800 10100

Neutrophil percentage (%) 66.13 7.026 49.3 81.1

Lymphocyte percentage (%) 23.61 6.267 11.1 39.9

Monocyte percentage (%) 6.703 1.571 3.4 12.0

Eosinophil percentage (%) 2.877 1.961 0.0 5.5

Basophil percentage (%) 0.683 0.373 0.1 1.6

WBC, white blood cell; SD, standard deviation.

**Table 3c. Characteristics of leukocyte differentials in the female NF1 group**

Mean value SD Minimum value Maximum value

WBC count (/µL) 6451 2029 3100 13100

Neutrophil percentage (%) 66.25 8.101 43.6 82.2

Lymphocyte percentage (%) 24.58 7.248 10.7 48.4

Monocyte percentage (%) 6.163 1.511 3.5 11.6

Eosinophil percentage (%) 2.310 2.082 0.0 12.7

Basophil percentage (%) 0.702 0.343 0.1 2.5

WBC, white blood cell; SD, standard deviation.
