# Supplementary material for: Systemic skewing of peripheral blood leukocyte composition in neurofibromatosis type 1
Source: Front Immunol. 2026 Jun 30;17:1849927. doi: 10.3389/fimmu.2026.1849927 (PMC13364682; doi:10.3389/fimmu.2026.1849927)
Supplement: Supplementary file 8 [file Table5.docx]

**Supplementary Table 5. Sex differences in raw data of control patients for leukocyte differentials**

Median value

Male (n = 50) Female (n = 37) *P*-value

WBC count (/µL) 6000 5600 0.381

Neutrophil percentage (%) 56.20 59.00 0.381

Lymphocyte percentage (%) 33.20 30.70 0.463

Monocyte percentage (%) 6.450 54.00 0.027

Eosinophil percentage (%) 2.650 28.00 0.696

Basophil percentage (%) 0.400 0.500 0.381

WBC, white blood cell.
