# Supplementary material for: Systemic skewing of peripheral blood leukocyte composition in neurofibromatosis type 1
Source: Front Immunol. 2026 Jun 30;17:1849927. doi: 10.3389/fimmu.2026.1849927 (PMC13364682; doi:10.3389/fimmu.2026.1849927)
Supplement: Supplementary file 9 [file Table6.docx]

**Supplementary Table 6a. Comparison of raw leukocyte differential data between combined control and NF1 subjects**

Median value

Control NF1 *P*-value

White blood cell count (/µL) 5800 6100 0.048

Neutrophil percentage (%) 57.30 66.70 <0.001

Lymphocyte percentage (%) 32.90 23.70 <0.001

Monocyte percentage (%) 5.900 6.100 0.080

Eosinophil percentage (%) 2.700 2.000 0.001

Basophil percentage (%) 05.00 0.700 0.002

**Supplementary Table 6b. Comparison of raw leukocyte differential data between control and NF1 males**

Median value

Control NF1 *P*-value

White blood cell count (/µL) 6000 6000 0.405

Neutrophil percentage (%) 56.20 66.80 <0.001

Lymphocyte percentage (%) 33.20 23.10 <0.001

Monocyte percentage (%) 6.450 6.450 0.405

Eosinophil percentage (%) 2.650 2.300 0.298

Basophil percentage (%) 0.400 0.650 0.033

**Supplementary Table 6c. Comparison of raw leukocyte differential data between control and NF1 females**

Median value

Control NF1 *P*-value

White blood cell count (/µL) 5600 6100 0.042

Neutrophil percentage (%) 59.00 66.70 <0.001

Lymphocyte percentage (%) 30.70 24.10 <0.001

Monocyte percentage (%) 5.400 5.800 0.010

Eosinophil percentage (%) 2.800 1.700 0.010

Basophil percentage (%) 0.500 0.700 0.157
