# Supplementary material for: Systemic skewing of peripheral blood leukocyte composition in neurofibromatosis type 1
Source: Front Immunol. 2026 Jun 30;17:1849927. doi: 10.3389/fimmu.2026.1849927 (PMC13364682; doi:10.3389/fimmu.2026.1849927)
Supplement: Supplementary file 10 [file Table7.docx]

**Supplementary Table 7. Associations between NF1 phenotypes and parameter Z-scores**

WBC-c Neu-p Lym-p Mono-p Eo-p Ba-p

MV for D-classes

D1 0.446 0.794 −1.154 0.649 −0.108 0.147

D2 0.106 0.857 −0.943 0.232 −0.683 0.261

D3 0.039 1.150 −1.146 0.349 −0.532 −0.082

D4 0.023 0.991 −1.024 0.349 −0.609 0.384

*P*-value 0.830 0.830 0.830 0.830 0.830 0.830

MV for N-classes

N0 0.132 1.183 −1.159 0.232 −0.562 0.032

N1 −0.295 1.345 −1.599 0.280 −0.518 −0.082

N2 0.677 1.169 −1.159 0.349 −0.436 0.147

*P*-value 0.153 0.538 0.538 0.538 0.538 0.614

MV for B-classes

B0 0.132 0.963 −1.084 0.345 −0.571 0.147

B1 −0.026 1.117 −1.033 0.573 −0.573 0.204

B2 0.027 0.933 −0.872 0.215 −0.686 0.147

*P*-value 0.922 0.922 0.922 0.922 0.922 0.922

MV, median value; WBC-c, white blood cell count; Neu-p, neutrophil percentage; Lym-p, lymphocyte percentage; Mono-p, monocyte percentage; Eo-p, eosinophil percentage; Ba-p, basophil percentage.
