# Supplementary material for: Systemic skewing of peripheral blood leukocyte composition in neurofibromatosis type 1
Source: Front Immunol. 2026 Jun 30;17:1849927. doi: 10.3389/fimmu.2026.1849927 (PMC13364682; doi:10.3389/fimmu.2026.1849927)
Supplement: Supplementary file 11 [file Table8.docx]

**Supplementary Table 8. Sex differences in raw data of NF1 patients for leukocyte differentials**

Median value

Male (n = 78) Female (n = 147) *P*-value

White blood cell count (/µL) 6000 6100 0.919

Neutrophil percentage (%) 66.80 66.70 0.919

Lymphocyte percentage (%) 23.10 24.10 0.796

Monocyte percentage (%) 6.450 5.800 0.020

Eosinophil percentage (%) 2.300 1.700 0.019

Basophil percentage (%) 0.650 0.700 0.796
